# Influence of Lattice Mismatch on Structural and Functional Properties of Epitaxial Ba_0.7_Sr_0.3_TiO_3_ Thin Films

**DOI:** 10.3390/ma16176036

**Published:** 2023-09-02

**Authors:** Jonas Wawra, Kornelius Nielsch, Ruben Hühne

**Affiliations:** 1 Leibniz Institute for Solid State and Materials Research, Helmholtzstrasse 20, D-01069 Dresden, Germany; j.wawra@ifw-dresden.de (J.W.); k.nielsch@ifw-dresden.de (K.N.); 2 Institute for Applied Physics, TUD Dresden University of Technology, D-01062 Dresden, Germany

**Keywords:** ferroelectrics, phase transition, relaxor, epitaxy, barium strontium titanate, thin films, pulsed laser deposition

## Abstract

Substrate-induced strains can significantly influence the structural properties of epitaxial thin films. In ferroelectrics, this might lead to significant changes in the functional properties due to the strong electromechanical coupling in those materials. To study this in more detail, epitaxial Ba${}_{0.7}$Sr${}_{0.3}$TiO${}_{3}$ films, which have a perovskite structure and a structural phase transition close to room temperature, were grown with different thicknesses on *RE*ScO${}_{3}$ (*RE*–rare earth element) substrates having a smaller lattice mismatch compared to SrTiO${}_{3}$. A fully strained SrRuO${}_{3}$ bottom electrode and Pt top contacts were used to achieve a capacitor-like architecture. Different X-ray diffraction techniques were applied to study the microstructure of the films. Epitaxial films with a higher crystalline quality were obtained on scandates in comparison to SrTiO${}_{3}$, whereas the strain state of the functional layer was strongly dependent on the chosen substrate and the thickness. Differences in permittivity and a non-linear polarization behavior were observed at higher temperatures, suggesting that ferroelectricity is supressed under tensile strain conditions in contrast to compressive strain for our measurement configuration, while a similar reentrant relaxor-like behavior was found in all studied layers below 0°C.

## 1. Introduction

Whereas ferroelectric oxide thin films are already used in many modern devices, research has been focusing over the last few years on exploiting their properties for novel applications [1]. For example, their opto-electronic properties are tested in high-frequency modulators [2,3,4], while the excellent dielectric and switching characteristics are promising for energy storage purposes [5,6]. Additionally, the pyroelectric properties might be beneficial for energy-harvesting devices as well as for the use in eco-friendly and efficient solid-state cooling [7,8]. The materials showing the highest potential are lead-containing relaxor solid solutions, which reveal extraordinary electromechanical responses. The relaxor-like behavior is described by the appearance of nanoscale regions, resulting in a spontaneous polarization even at high temperatures [9]. This feature is often explained by the chemical disorder in the system [10], but unfortunately, the underlying mechanism leading to the relaxor behavior remains unclear [11,12]. Additionally, the high toxicity of lead is detrimental; therefore, major efforts are made to develop lead-free alternatives [13]. One of those is the well-known perovskite ferroelectric barium titanate BaTiO${}_{3}$. While its properties are inferior for many applications in comparison to its lead-containing counterparts, the substitution of cations is an effective way to create solid solutions in order to enhance the ferroelectric characteristics [14,15,16,17]. Among them is Ba${}_{1-x}$Sr${}_{x}$TiO${}_{3}$, which is commonly used in varactors [18,19] due to its high dielectric constant and tunability [20]. The replacement of Ba${}^{2+}$ with smaller Sr${}^{2+}$ results in a stabilization of the higher symmetric perovskite phases and therefore reduces the phase transition temperatures with increasing substitution *x* [21] compared to pure BaTiO${}_{3}$. Additionally, many studies report that the type of phase transition in this material shifts from a classic ferroelectric transition to a diffuse or relaxor behavior with increasing Sr content [22,23]. This is consistent with our own studies on epitaxial Ba${}_{1-x}$Sr${}_{x}$TiO${}_{3}$ films, where a relaxor-like transition was observed in the frequency-dependent permittivity on samples with a composition of Ba${}_{0.7}$Sr${}_{0.3}$TiO${}_{3}$ (BST) [24]. Since the strain induced by the substrate can alter the properties of epitaxial thin films as demonstrated by theoretical calculations [25,26] as well as experimental investigations [27,28], the choice of substrate material offers an easy way to modify ferroelectric films. However, the precise control of the strain in an epitaxial thin film is usually not straightforward, in particular for samples exceeding the critical thickness, which can be as low as a few nm only in some systems [29]. If the film thickness increases further in such a case, the formation of dislocations is observed, resulting in a relaxation during growth. The mechanical strain in these film is then not only a function of the difference between the thermal expansion coefficient of film and substrate but also of the deposition temperature, thickness and the dislocation density [25], where some factors can be hard to quantify. To study the effect of strain on the prototypical lead-free solid solution BST, epitaxial films on *RE*ScO${}_{3}$ (*RE* is a rare earth element) substrates were prepared, which have a much smaller lattice mismatch in comparison to the previously used SrTiO${}_{3}$ (STO). This allows growing films with a higher critical thickness as well as preparing samples with equal thicknesses but different strain states, which will be used to compare their electrical properties.

## 2. Materials and Methods

SrRuO${}_{3}$ (SRO) and BST layers were grown on different single crystal substrates (CrysTec GmbH, Berlin, Germany) by a standard on axis pulsed laser deposition (PLD) process, using a KrF laser with a wavelength of λ=248 nm. The laser was focused as a rectangular spot with a size of $A=3\;mm^{2}$ on stoichiometric polycrystalline targets, giving an energy density of $w=1\;J/cm^{-2}$. The SRO target was homemade from polycrystalline powder, whereas the BST target was prepared at Fraunhofer IKTS Dresden by a conventional solid-state reaction method. During the deposition, the oxygen background pressure was kept at $p_{O_{2}}=0.01\;mbar$, whereas the distance between the target and substrate was set to 50 mm. First, an SRO buffer layer with a thickness of 20 nm was deposited at a substrate temperature $T_{S}=750\;{}^{\circ}C$ and a laser frequency of f=3Hz. Next, the temperature was increased to $T_{S}=900\;{}^{\circ}C$ to deposit the BST layer with a frequency of f=5 Hz. Subsequently, the sample was cooled back down to room temperature and removed from the vacuum chamber.

Since the deposition rate of both layers was already known from previous studies [24], the number of pulses could be adjusted to reach the desired thickness. A Bruker DektakXT stylus profilometer (Bruker, Billerica, MA, USA) was used to check the thickness after the growth and no significant variation in dependence of the used substrate was found. Surface morphology measurements were carried out using a Bruker Icon atomic force microscope (AFM) in tapping mode.

Afterwards, a photolithographically prepared shadow mask was put on the sample surface. Through this mask, 5 nm Ti as well as 100 nm Pt were sputtered on the surface of the samples at room temperature to create round electrodes with a radius of r=100 μm. Later, the shadow mask was removed by a standard lift-off process.

The microstructure of the samples was characterized by X-ray diffraction (XRD) utilizing a Bruker D8 Advance with Co-K${}_{\alpha }$ radiation for the 2θ-θ scans. Additionally, a Philips X’Pert four-cycle goniometer (Malvern Panalytical, Almelo, The Netherlands) equipped with a Cu tube and a primary Ge monochromator was used to perform reciprocal space map (RSM) and rocking curve measurements.

The electrical characterization of the chosen samples was performed by contacting two probes to the Pt electrodes and applying a field along the out-of-plane direction through the whole film thickness, using the SRO buffer layer as a bottom electrode. In this setup, the capacitance *C* could be measured by an HP 4284 precision LCR Meter (Keysight, Santa Rosa, CA, USA) using an alternating voltage signal with a frequency between 0.1 and 10 kHz with 50 mV amplitude. Afterwards, the relative permittivity was calculated by assuming two equal plate capacitors in series using $\varepsilon_{r}=C\;\cdot \;2d/\left(\varepsilon_{0}\;\cdot \;\pi r^{2}\right)$, with the vacuum permittivity $\varepsilon_{0}$, the film thickness *d* and the area of the Pt contact $\pi r^{2}$. Polarization measurements were carried out with a Radiant Technologies Multiferroic tester (Radiant Technologies, Inc., Albuquerque, NM, USA) in the same contact configuration. For the electric field-dependent loops, a triangular profile with a frequency of 1 kHz was used. First, a positive pulse was applied to generate a defined polarization state in the film before a whole loop was measured anti-clockwise. All electrical characterizations were performed in a Linkam LTSE420-P probe stage (Linkam Scientific Instruments Ltd., Redhill, UK) that allowed for temperature-dependent measurements while cooling down the sample from 250 °C to −150 °C.

## 3. Results

The mostly used parameter to quantify the strain in hetero-epitaxial layer architectures is the lattice mismatch $\alpha =\left(a_{s}-a_{f}\right)/a_{s}$ between the in-plane lattice parameter of the substrate $a_{s}$ and the film $a_{f}$. STO is a cubic perovskite material with the space group *Pm$\bar{3}$m* (no. 221). Its lattice parameter is $a_{\mathrm{STO}}=3.905\;Å$, which leads to a mismatch of −1.9% for bulk BST, that crystallizes in the same space group with a lattice parameter of $a_{\mathrm{BST}}=3.980\;Å$ at room temperature. In bulk, a structural phase transition to the ferroelectric tetragonal structure with the space group *P4mm* (no. 99) is observed slightly below room temperature. As in pure BaTiO${}_{3}$, the ferroelectricity in this phase originates from the spontaneous polarization caused by the shift of position between the positively charged Ti${}^{4+}$ cation and the negatively charged O${}^{2-}$ oxygen octahedra. Since there are no other cubic oxide single crystals with a smaller mismatch to BST available, orthorhombic *RE*ScO${}_{3}$ single crystals were chosen as an alternative, which crystallize in the space group *Pbnm* (no. 62). If cut along the (1 1 0)-plane, a rectangular surface structure is obtained. The lattice parameters of the almost quadratic surface unit cell with $a_{1\bar{1}0}$ parallel to the [1 $\bar{1}$ 0] direction and $a_{001}$ parallel to the [0 0 1] can be calculated from the orthorhombic lattice parameters *a*, *b* and *c* with the relations $a_{1\bar{1}0}=0.5\sqrt{a^{2}+b^{2}}$ and $a_{001}=0.5c$ respectively. For our studies, DyScO${}_{3}$ (DSO), GdScO${}_{3}$ (GSO) and NdScO${}_{3}$ (NSO) were selected to achieve a broad range of mismatches, ranging from compressive to tensile strain conditions for BST layers. The lattice constants as well as the size of the surface unit cell of the used *RE*ScO${}_{3}$ crystals are summarized in Table 1.

From those lattice parameters, the mismatches along both in-plane directions to the bulk (1 0 0) BST lattice parameters are calculated that will lead to a biaxial strain state. The results are summarized for all substrates in Table 2.

### 3.1. Structural Characterization

**Figure 1:**
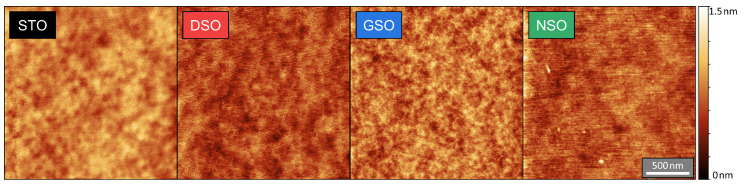
AFM scans of 250nm BST layers on different SRO-buffered substrates. From left to right: STO, DSO, GSO and NSO.

Before the top contacts were prepared, AFM scans of the BST/SRO heterostructure surface were taken; the results are shown in Figure 1. In all cases, a dense and smooth surface without any notable features is observed; i.e., no significant influence of the substrate could be identified. The root-mean-square roughness was between 130 and 170 pm for all films studied, which is comparable to the results of previous studies [24].

**Figure 2:**
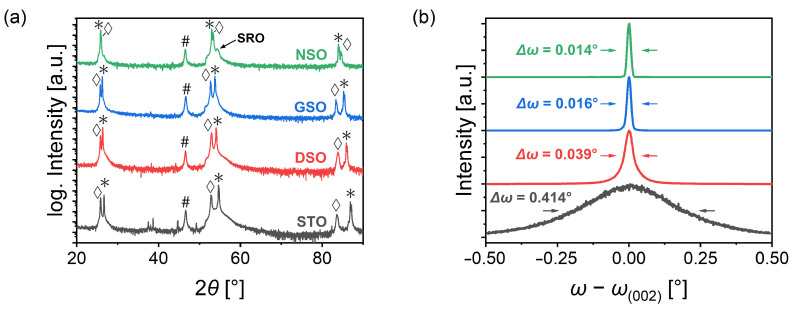
(**a**) 2θ-θ XRD scans of 250 nm BST layer architectures on different substrates. Corresponding substrate peaks are marked with *, (0 0 *l*) BST peaks are marked with ⋄ and Pt peaks are marked with #. (**b**) Rocking curves for the same samples.

Figure 2a shows 2θ-θ scans for the complete Pt/BST/SRO architectures on the different substrates. All BST films had a thickness of roughly 250 nm. In all samples, sharp peaks with high intensity are visible next to the out-of-plane reflexions of the substrates, which can be indexed with the (0 0 *l*) planes of the BST structure, confirming the successful c-axis-oriented growth. This peak is slightly shifted toward lower angles compared to the corresponding bulk peak for the films on STO, DSO and GSO, which corresponds to a larger out-of-plane lattice parameter indicating a tetragonal distortion of those layers. Due to the tensile strain condition on NSO, this shift is not observed in the corresponding scan. The structure on NSO also shows the pseudo-cubic (0 0 2) reflection of the SRO buffer layer, which is not visible for the other spectra, due to the small difference between the pseudo-cubic lattice parameter of SRO ($a_{\mathrm{SRO}(\mathrm{pc})}=3.929\;Å$) and the substrate values as well as the small layer thickness. The peak observed in all scans at 2θ=46.7° results from the (1 1 1) planes of the Pt electrodes.

**Figure A1:**
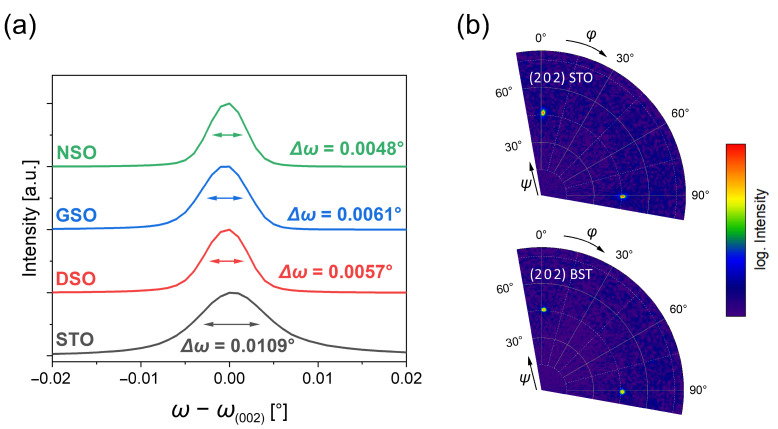
(**a**) Rocking curve scans of the four different substrates. (**b**) Texture measurement of the (2 0 2) planes of a STO single crystal substrate (**top**) and the corresponding BST layer (**bottom**).

To obtain more detailed information on the crystalline quality of the BST-films, (0 0 2) rocking curve scans were measured for these samples. The results are shown in Figure 2b. It is clearly visible that the films on the *RE*ScO${}_{3}$ substrates show significant smaller full width at half maximum (FWHM) Δω values compared to the film on STO. *RE*ScO${}_{3}$ crystals are grown with a higher quality using the Czochralski method in comparison to STO crystals, which are grown via the Verneuil method [30,31]. However, rocking curve scans of the substrates (Figure A1a in Appendix A) revealed a much smaller difference in the FWHM values ( 0.006 ° for the *RE*ScO${}_{3}$ substrates vs. 0.011 ° for STO). We assume that the higher quality of the *RE*ScO${}_{3}$ substrates in combination with the smaller lattice mismatch significantly reduced the mosaicity on the films compared to the sample on STO (see RSM measurements below).

**Figure 3:**
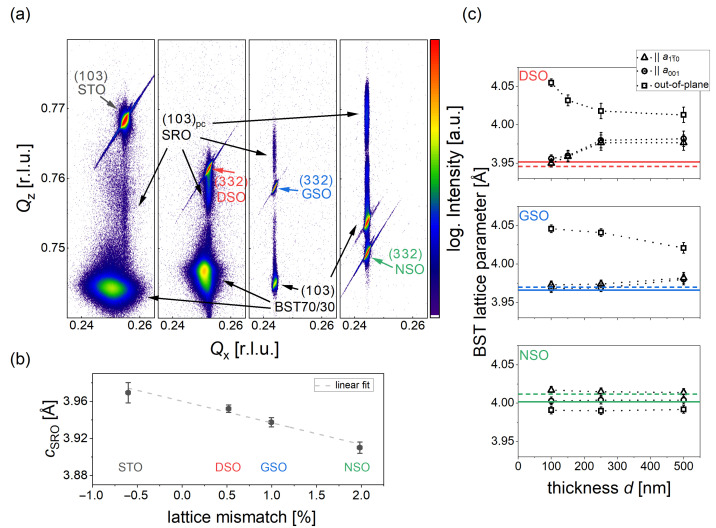
(**a**) RSMs around the (1 0 3) BST ( 250 nm) peak on the different substrates. From left to right: STO, DSO, GSO, NSO. (**b**) Out-of-plane lattice parameter of the SRO bottom electrode in dependence of the lattice mismatch induced by the different substrates. (**c**) Thickness dependence of the BST lattice parameters on the different *RE*ScO${}_{3}$ substrates. From top to bottom: DSO, GSO, NSO. The colored lines represent the literature value for the pseudo-cubic lattice parameter of the corresponding crystal along the [0 0 1] direction and the dashed colored line along the [1 $\bar{1}$ 0] direction.

XRD texture measurements (Figure A1b in Appendix A) confirmed a pure epitaxial growth on all substrates. Detailed RSM measurements were performed to acquire additional insights on the quality of our samples and to calculate the in- and out-of-plane lattice parameters of the different layers. Exemplarily, the scans around the (1 0 3) BST peak are shown for each substrate in Figure 3a. The highest intensities peaks originate from the corresponding substrates. As expected, those peaks shift to lower $Q_{x}$ and $Q_{z}$ values due to the increasing unit cell parameters from STO to NSO.

The weak reflections elongated along the $Q_{z}$ direction can be assigned to the (1 0 3) pseudo-cubic planes of the thin SRO buffer layer. No broadening or shift along the $Q_{x}$ direction is observed, demonstrating that the in-plane lattice parameters of the substrate are transferred to the SRO layer. The center of the SRO peaks was used to calculate the out-of-plane lattice parameter $c_{\mathrm{SRO}}$ in dependence of the average lattice mismatch to the corresponding substrates, using the literature pseudo-cubic bulk value of 3.929 *Å*. The results are shown in Figure 3b. A decrease in the out-of-plane lattice parameter with increasing mismatch is observed due to of a fully strained growth. A small increase in the pseudo-cubic unit cell of roughly 2% is observed. This is usually associated with small losses of the volatile RuO${}_{2}$ in the SRO layer due to the high deposition temperatures and the low oxygen background pressure during PLD growth, as reported by other groups [32,33]. However, the RuO${}_{2}$ losses seem to be relatively small as higher deficiencies would lead to the formation of the Ruddlesden–Popper phase Sr${}_{3}$Ru${}_{2}$O${}_{7}$ [34], which would lead to an even larger increase in the out-of-plane lattice parameter or an additional diffraction peak, which was not observed in any of our samples.

In all four RSM maps, the BST peak is clearly visible; however, the shape and position show notable differences. Similar to the rocking curve measurements, the film on STO shows a broad peak, which is elongated in the $Q_{x}$ direction At the same time, the maximum is at a lower $Q_{x}$ value compared to the substrate reflex. This indicates that dislocations are formed early during layer growth, which results in a relaxed strain state with a high mosaicity resulting in the broad peak. A more narrow spot along $Q_{x}$ is observed on DSO; instead, the peak is elongated along the $Q_{z}$ direction. Accordingly, dislocation might be introduced at a later point during growth, which indicates a coherent growth of the first layers on SRO before strain relaxation starts. In comparison, the film peaks on GSO and NSO show nearly no broadening; i.e., a fully strained growth case is observed in both cases. This is consistent with the sharpest rocking curve for these samples (compare Figure 2b). A strong shift to a higher $Q_{z}$ value is found for the film on NSO due to the much smaller out-of-plane lattice parameter, originating from the tensile strain state due to the large in-plane lattice parameter of the substrate.

To study the influence of the BST layer thickness, additional films were grown with different pulse numbers and studied with RSM measurements. The position of the maximum intensity from the (1 0 3), (0 1 3), (0 $\bar{1}$ 3) and ($\bar{1}$ 0 3) BST reflexes were used to determine the lattice parameters [35], which are summarized in Figure 3c. For DSO substrates, a strong dependence on thickness was found for thinner samples. In particular, the thinnest film shows equal in-plane lattice parameters as the substrate, pointing to a fully strained state of our BST layers up to a thickness of 100 nm on DSO. If the thickness increases further, the in-plane lattice parameters become larger, whereas the out-of-plane lattice parameter decreases due to relaxation processes during growth. For about 500 nm thick layers, almost no difference was found for the lattice parameters in comparison to the 250 nm thick film. For films on GSO, a similar behavior was observed; however, the relaxation process starts at a higher thickness compared to DSO, which is expected due to the smaller lattice mismatch. Therefore, only the 500 nm thick film shows larger in-plane lattice constants than the substrate as well as a slight decrease in the out-of-plane parameter. Interestingly, all films on NSO show roughly the same lattice parameters, and no indication for relaxation was found up to a thickness of 500 nm despite the fact that the lattice mismatch is larger than for films on GSO. In this case, even an orthorhombic structure can be assumed, as all lattice parameters are different from each other.

### 3.2. Electrical Characterization

**Figure 4:**
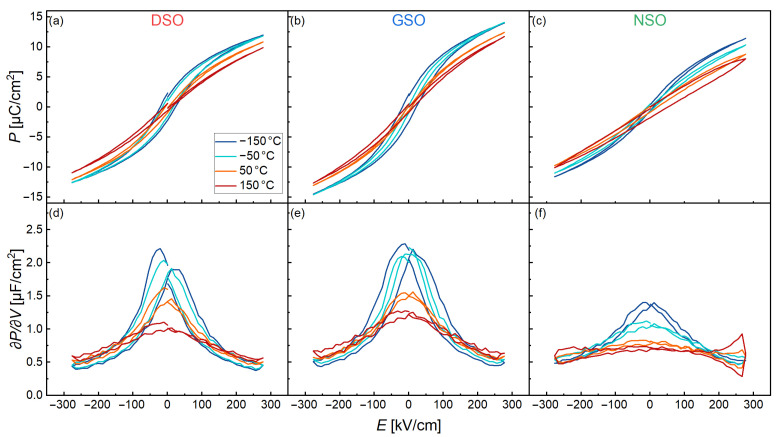
P(E) loops for Pt/BST/SRO layer architectures with a dielectric layer thickness of 250 nm on (**a**) DSO, (**b**) GSO and (**c**) NSO. The corresponding derivative ∂P/∂V are shown in (**d**–**f**), respectively.

To study the ferroelectric behavior of the grown BST layer architectures, the dependence of the polarization *P* on the electric field *E* was measured at different temperatures. Selected results are shown for the 250 nm films in Figure 4a–c.

With decreasing temperature, the remnant and saturation polarization $P_{r}$ and $P_{s}$, as well as the coercive field $E_{c}$ increase for all samples, and S-shaped hysteresis loops are observed at low temperatures. On DSO, the increase in $P_{r}$ and $P_{s}$ is continuous when cooling the sample down from 150 to −50 °C, while no major change is observed when cooling further down to −150 °C. The structure on GSO shows a small change of the hysteresis loop between 150 and 50 °C, whereas a significantly higher $P_{r}$ value is observed during cooling toward −50 °C. At lower temperatures, an additional increase in $P_{r}$ and $E_{c}$ was found for this sample. Overall, the film architecture grown on GSO shows a higher $P_{s}$ compared to the sample on DSO, which might be a result of the higher tetragonal distortion due to the fully strained growth on GSO, as discussed in the previous section.

The sample on NSO, which showed lower out-of-plane than in-plane parameters, reveals lower polarizations values as the films on the other substrates. Even at −150 °C, this film architecture results in a much slimmer polarization loop compared to the ones on DSO and GSO. While the S-shape of the loop is still identifiable at high temperatures for the samples grown on DSO and GSO, an almost linear P(E) dependence is measured for the film on NSO above 50 °C. This behavior is even better visible, when the derivative of the polarization ∂P/∂V (which is equal to the capacitance per unit area) is plotted versus the applied field as shown in Figure 4d–f. Here, the non-linear behavior with two peaks around the coercive field demonstrates the ferroelectric switching of our capacitors. In case of films on NSO, the ∂P/∂V curves become nearly field independent at higher temperatures, indicating that the ferroelectricity is vanishing. Instead, the curve at 50 °C shows a hysteresis at higher positive fields, which is a typical sign when leakage has a notable contribution to the corresponding P(E) loop. This feature increases at 150 °C, suggesting that leakage increase with temperature.

**Figure 5:**
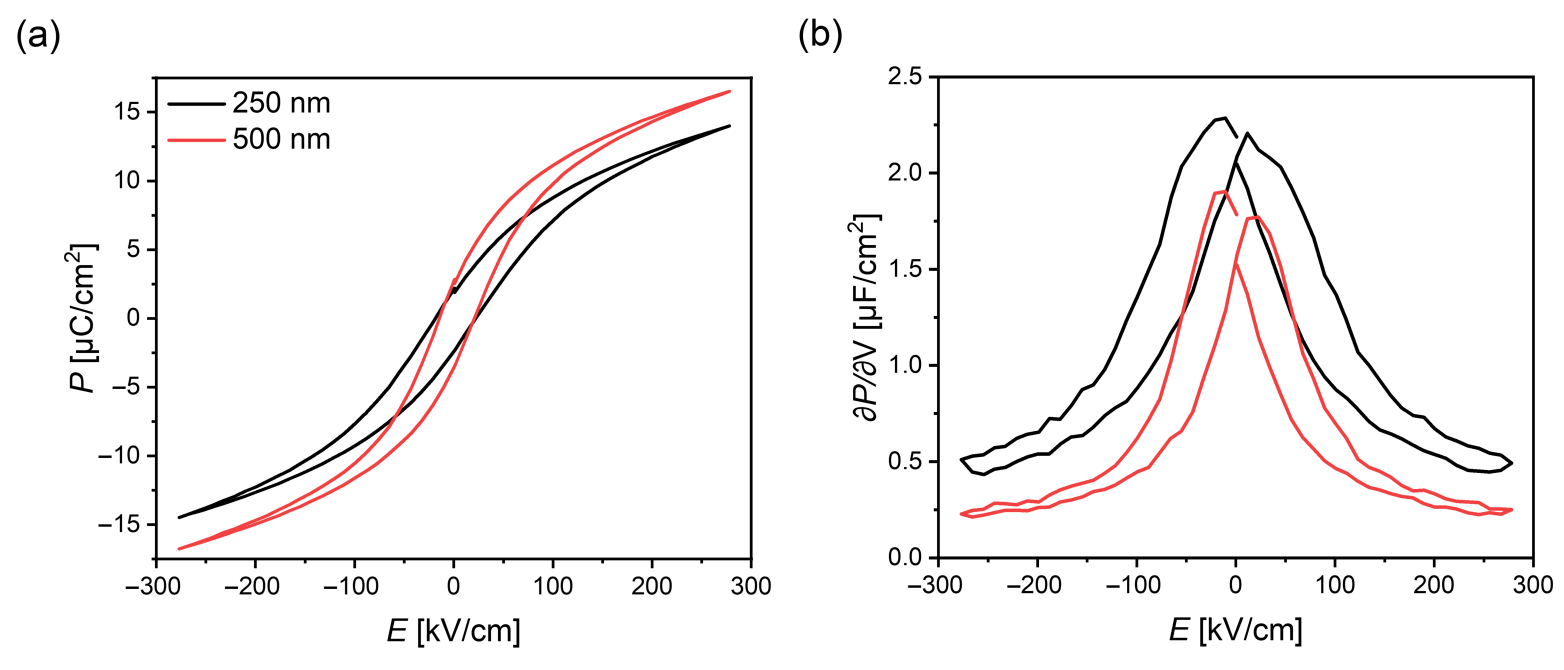
Comparison of the (**a**) P(E) loops and (**b**) its derivative ∂P/∂V at −150 °C of a film architecture with a 250 nm or 500 nm BST layer, respectively, grown on GSO.

Since a strong dependence of *P* on the out-of-plane lattice parameter was found for the about 250 nm thick samples on the different *RE*ScO${}_{3}$ substrates and a comparable decrease was found for the out-of plane lattice parameter when increasing the BST layer thickness to 500 nm on GSO, the P(E) loops of those two samples were compared. The results are shown for the lowest temperature in Figure 5a as an example. While the coercive field shows almost no change, the polarization values increase despite the smaller out-of-plane lattice parameter of the thicker layer. This behavior might be explained with a reduced clamping to the substrate, which allows larger parts of the film to align along the applied field [36,37], which in turn leads to a higher measured polarization. Again, the derivative of the polarization loops was calculated and compared as shown in Figure 5b. The main difference in those curves is an additional offset along the ∂P/∂V-axis, indicating a different linear contribution for the capacitance. Here, the offset is roughly half the value as the thickness of the BST layer doubles. In ferroelectric capacitors, the linear component is often associated with interface layers that show significantly reduced functional properties, often referred to as “dead layers” [38,39], and therefore contribute as a linear dielectric offset to the ferroelectric non-linear capacitance. This linear behavior does not change in dependence of the substrate, as shown in Figure 4d–f for the samples with a layer thickness of about 250 nm. The thicker the ferroelectric film becomes, the lower the relative contribution of the interfaces and therefore the linear contribution, which might offer an explanation for the observed behavior.

**Figure 6:**
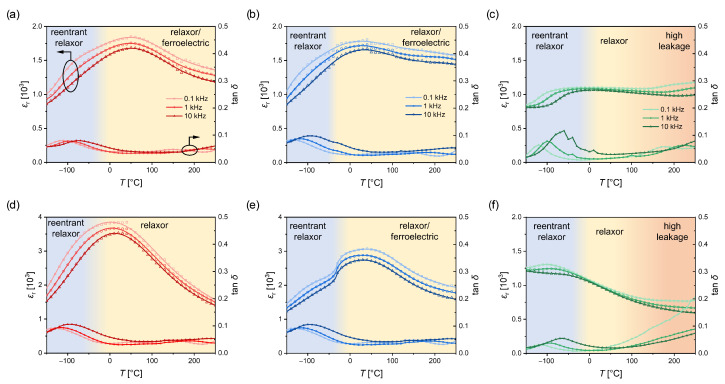
Real part of the relative permittivity $\varepsilon_{r}$ and dielectric loss tanδ as a function of temperature *T* at different frequencies of film architectures with a 250 nm and 500 nm BST layer on (**a**,**d**) DSO, (**b**,**e**) GSO and (**c**,**f**) NSO.

To acquire a more comprehensive picture of the temperature-dependent dielectric behavior of our layer architectures, the low field permittivity was measured at different frequencies. The results are shown in Figure 6. In the following, the results will be discussed first for the about 250 nm thick films (i.e., Figure 6a–c). The observed dependencies can be divided roughly into different temperature regimes. At high temperatures (i.e., above 100 °C), the measured permittivity is frequency dependent for all samples, which is unexpected for a conventional ferroelectric material. We assume that this frequency dependence is a result of the high leakage in the architectures grown on NSO, as already discussed for the hysteresis loops (compare Figure 4). As the leakage is typically reduced with decreasing temperatures, the permittivity becomes less frequency dependent at medium temperatures between 125 and 0 °C. Since no notable contributions from leakage were observed in the hysteresis loops even at 150 °C for the films on DSO and GSO and the frequency dependence is still observed for the medium-temperature range, it might have another origin in these samples. It should be noted at this place that a similar behavior is often observed in measurement configurations with a poorly conducting electrode [40]. While SRO is usually considered as a good conducting oxide, its resistivity is significantly higher compared to a pure metal like Pt and might be responsible for the observed behavior. It was shown that tensile strain leads to an increase in the ferromagnetic Curie temperature in SRO [41], which results in a higher conductivity and explains why this effect is reduced in the structure on NSO where the highest tensile strain is induced into the oxide electrode layer. A low conductivity of the bottom electrode might also lead to a reduction in permittivity [42]; therefore, our results should be interpreted with caution. Nonetheless, the $\varepsilon_{r}$ values are considerably higher on DSO and GSO when compared to NSO, where the conduction of the oxide layer seems to have less impact, indicating that compressive strain increase the permittivity in comparison to the tensile strain. Since compressive strain typically favors the dielectric dipoles in perovskite ferroelectrics to order along the out-of-plane direction [25], higher depolarization fields are expected, which lead to a higher $\varepsilon_{r}$. Additionally, the layers on DSO and GSO show, in contrast to NSO, a broad peak for the maximum permittivity at T=50 °C and T=40 °C, respectively.

A frequency-dependent peak is typically observed in relaxor ferroelectrics due to an increasing size or ordering of polar regions inside the material with decreasing temperature up to a certain point $T_{m}$. The absence of such a peak for films on NSO might originate from the tensile strain leading to a preferred polarization along the in-plane direction [43]. Therefore, the increased polar order is barely visible in measurements along the out-of-plane direction, as in our case. Nonetheless, a frequency-dependent decrease in permittivity is observed in those samples at low temperatures between −20 and −80 °C. At the same time, peaks are observed for the dielectric loss tanδ at the same temperatures. Such a phase shift between the small amplitude signal and the response of the dielectric film at a point, where the permittivity decreases, is often observed in materials, which show a reentrant relaxor behavior. It is associated with a reduced mobility or freezing of the growing polar regions. At a certain point, those materials go into a state similar to a spin glass, where the dielectric dipoles are randomly aligned, and no reorientation is possible with small fields [44]. Even though the loss peaks are slightly smaller, less frequency dependent and the reduction in permittivity is less significant due to the broad peak above room temperature, a similar behavior is observed on DSO and GSO in the same temperature range. As demonstrated by the polarization measurements, the hysteresis loops reveal an increase in the coercive field and the polarization values, indicating a transition from the more relaxor-like behavior to a classic ferroelectric.

Similar permittivity measurements were performed for the layer architectures with a BST thickness of about 500 nm. The results are summarized in Figure 6d–f. A significant increase in permittivity is found for the films on DSO and GSO (note the different scale). Again, the mechanical boundary conditions resulting from clamping on the substrate surface might explain this observation. With increased layer thickness, these effects are less prominent due to relaxation effects. Since the critical thickness of the BST layer is significantly lower on DSO compared to GSO, such relaxation effects might have a larger impact on the films on DSO, resulting in a higher permittivity increase. In contrast, no significant increase is observed for thicker BST layers on NSO, which is consistent with the fact that no relaxation was observed even for the 500 nm thick film. It should be also noticed that the layers on DSO show a more relaxor-like behavior in comparison to all other films, as $T_{m}$ slightly shifts to lower temperatures with decreasing frequency. While this is not clearly visible for the films on GSO, a more pronounced change in slope of $\varepsilon_{r}$ is observed in the low-temperature range. For all three layer architectures, the dielectric loss shows a similar behavior as for the thinner BST layers, indicating similar reentrant-like features at low temperatures.

## 4. Conclusions

In summary, the use of *RE*ScO${}_{3}$ substrates improved the crystalline quality of the epitaxial layer architectures in comparison to the previously studied STO and results in a coherent growth of the BST-SRO heterostructures up to thicknesses of over 100 nm. Polarization measurements revealed a ferroelectric behavior even at higher temperatures (up to 150 °C) under compressive strain, whereas a suppression of those properties was found under tensile strain. Overall, the slim polarization loops as well as the permittivity measurements indicate a relaxor-like behavior at temperatures above 50 °C, whereas a reentrant-like transition below 0 °C is observed under tensile and compressive strain. Additionally, the electrode material, interfaces and clamping effects might have a significant influence on the measurements, as indicated by the comparison of structures with a thicker dielectric layer. A temperature-dependent structural characterization by X-ray diffraction might help to further understand how the strain influence the properties of the prepared dielectric layers.

## Figures and Tables

**Table 1:** Lattice parameters *a*, *b* and *c* of the orthorhombic unit cell and the calculated values of the surface unit cell of the (1 1 0) surface for the used *RE*ScO${}_{3}$ substrates. Data taken from [30].

Crystal	*a*/Å	*b*/Å	*c*/Å	$a_{1\bar{1}0}$/Å	$a_{001}$/Å
DSO	5.440	5.704	7.903	3.946	3.952
GSO	5.480	5.746	7.932	3.970	3.966
NSO	5.575	5.776	8.003	4.014	4.002

**Table 2:** Calculated lattice mismatch α in % at room temperature between both in-plane directions of the substrates and the cubic BST.

	STO	DSO		GSO		NSO	
	a	$a_{1\bar{1}0}$	$a_{001}$	$a_{1\bar{1}0}$	$a_{001}$	$a_{1\bar{1}0}$	$a_{001}$
BST	−1.92	−0.86	−0.71	−0.25	−0.35	0.85	0.55

## Data Availability

All data in this work are available on request by contact with the corresponding author.
